# Role of women empowerment on mental health problems and care-seeking behavior among married women in Nepal: secondary analysis of nationally representative data

**DOI:** 10.1007/s00737-024-01433-5

**Published:** 2024-02-05

**Authors:** Md Shajedur Rahman Shawon, Fariha Binte Hossain, Robin Ahmed, Isfar Jahan Poly, Moushumi Hasan, Mohammad Rifat Rahman

**Affiliations:** 1 Centre for Big Data Research in Health, University of New South Wales, Level 2, AGSM Building (G27), Sydney, Australia; 2 School of Population Health, University of New South Wales, Sydney, Australia; 3 Sir Salimullah Medical College, Dhaka, Bangladesh; 4 University of Chittagong, Chattogram, Bangladesh; 5 Dhaka, Bangladesh

**Keywords:** Women empowerment, Anxiety, Depression, Mental health, Care-seeking

## Abstract

**Purpose:**

This study investigates the associations between women empowerment and the prevalence of mental health symptoms and care-seeking behavior among ever-married Nepalese women aged 15–49 years.

**Methods:**

We utilized 2022 Nepal Demographic and Health Survey data to measure women empowerment, employing the Survey-Based Women’s Empowerment (SWPER) index. The index focuses on the domains of social independence, decision-making, and attitudes towards violence. Symptoms of anxiety and depression were measured using the Generalized Anxiety Disorder-7 scale (GAD-7) and the Patient Health Questionnaire (PHQ-9), respectively. Multiple logistic regression was performed to estimate adjusted odds ratio (aOR) for associations of women empowerment domains and mental health problems and care-seeking behavior.

**Results:**

Among 5556 women, the prevalence of symptoms of anxiety and depression was 23.1% and 6.1%, respectively. Among those with any symptoms of anxiety or depression, 18.3% sought care. Compared to women with low level of empowerment in the “social independence” domain, those with high level of empowerment were less likely to suffer from symptoms of anxiety (aOR = 0.68; 95%CI, 0.57–0.82) and depression (aOR = 0.69; 95%CI, 0.50–0.94). However, high empowerment in “decision-making” domain was associated with higher likelihood of anxiety (aOR = 1.67; 95%CI, 1.33–2.10) and depression (aOR = 1.80; 95%CI, 1.26–2.58). There was evidence of positive association between high empowerment in “decision-making” and care-seeking (aOR = 1.28; 95%CI, 0.96–1.71).

**Conclusions:**

This study underscores important roles of women empowerment on mental health symptoms and care-seeking behavior, suggesting the need to integrate empowerment initiatives into strategies to promote mental health among women in Nepal and similar low- and middle-income settings.

**Supplementary Information:**

The online version contains supplementary material available at 10.1007/s00737-024-01433-5.

## Introduction 

Women in low- and middle-income countries (LMICs) are disproportionately susceptible to common mental disorders compared to men, a vulnerability which can be attributed to a complex interplay of biological, social, and environmental factors (Patel 2007, Malhotra and Shah 2015, Rathod et al. 2017) . Social determinants play a pivotal role in the development of mental health problems in women (Compton 2015). Cultural impositions of rigid traditional roles that curtail personal liberties and lower social status can make women more prone to mental health issues. This susceptibility is further exacerbated by exposure to domestic and sexual violence (Srivastava 2012; Leight et al. 2022). Moreover, the pressures of multiple roles, gender discrimination, poverty, overwork, and other forms of abuse compound these challenges, negatively impacting women’s mental health across diverse cultures and countries (Malhotra and Shah 2015; Rai et al. 2021). The impact of social determinants of mental health often extends more broadly at the societal level. The key determinants recognized in a mental health promotion framework include social inclusion (social relationships, group activity involvement, civic engagement), freedom from discrimination and violence (diversity appreciation, physical security, self-determination), and access to economic resources (employment, education, housing, financial stability) (Walker et al. 2005) .

Empowerment significantly contributes to women’s ability to maintain health and access necessary health resources by facilitating effective navigation of social and economic environments (Leight et al. 2022). This leads to improved health behaviors and informed health-related decisions. Given the complexity of measuring women’s empowerment, especially in low- and middle-income countries, the Survey-Based Women’s Empowerment (SWPER) index was developed (Ewerling et al. 2017; Ewerling et al. 2020). This innovative tool, validated against the recognized Gender Development Index (GDI) and Gender Inequality Index (GII) (United Nations Development Programme 2016), offers a robust measure of women’s empowerment using Demographic and Health Surveys (DHS) data.

Nepal has a significant burden of mental health symptoms like anxiety and depression, with women suffering more than men (Rai et al. 2021, Nepal 2022). Despite Nepal’s commitment to achieving gender equality and women empowerment, a goal aligned with the Sustainable Development Goals (SDGs) launched in 2015, disparities and inequalities persist (UN Women). Considering the critical importance of women empowerment on mental health aspects of women, we aim to quantify the prevalence of symptoms of mental health problems and care-seeking behavior among Nepalese women and to investigate the associations of women empowerment with mental health problems and care-seeking behavior.

## Materials and methods

### Datasets and study design

This cross-sectional study used secondary data from the 2022 Nepal DHS (NDHS) (Ministry of Health and Population [Nepal] New ERA and ICF 2023), which included questions related to mental health and required information needed to calculate the SWPER index (Ewerling et al. 2020). The 2022 NDHS was executed by New ERA under the aegis of the Ministry of Health and Population, with technical assistance from ICF to the DHS Program, a US Agency for International Development (USAID) funded project (Ministry of Health and Population [Nepal] New ERA and ICF 2023). The 2022 NDHS received ethical approval from the Suaahara II, USAID’s integrated nutrition program. Before proceeding with the interview, each participant gave their informed written consent (Ministry of Health and Population [Nepal] New ERA and ICF 2023). We received anonymized dataset from the DHS website (https://dhsprogram.com/data/available-datasets.cfm) after submitting a research proposal, as per their guidelines.

The 2022 NDHS relied on a sampling frame derived from the updated version of the 2011 National Population and Housing Census (NPHC), supplied by the National Statistical Office (Ministry of Health and Population [Nepal] New ERA and ICF 2023). This sampling frame includes a comprehensive list of Nepal’s 36,020 sub-wards, representing the smallest administrative units considered in the survey. The 2022 NDHS utilized a two-stage stratified sample, with each of the seven provinces divided into urban and rural areas for stratification purposes. During the first stage, 476 primary sampling units (PSUs) were selected based on probability proportional to PSU size, with independent selection within each stratum of the sample allocation. A household listing operation was conducted in all the selected PSUs prior to the primary survey, and the resultant household list formed the sampling frame for the second stage of selection. From each cluster, thirty households were selected, leading to a total of 14,280 households. Large sub-wards, identified during the household listing operation and having over 300 households, were segmented, and then one segment was chosen for the survey proportional to segment size. Only preselected households were selected to be interviewed by the fieldworkers. To avoid bias, there were neither replacements nor alterations to the preselected households during the implementation stage. All women aged 15–49, either permanent residents or visitors who had stayed in the selected households the night before the survey, were eligible for interviewing, resulting in a 97.4% response rate (Ministry of Health and Population [Nepal] New ERA and ICF 2023). However, this analysis only included currently married women who have complete information on mental health variables and necessary data for calculating the SWPER index.

### Exposure variable: the SWPER index

To assess women empowerment, we used the validated and multidimensional SWPER index (Ewerling et al. 2017; Ewerling et al. 2020). The SWPER index leverages individual-level data from DHS surveys to assess relationships between women empowerment and various health outcomes. The SWPER index operationalizes women’s empowerment across three domains: attitude towards violence, social independence, and decision-making. The domain of “attitude towards violence” primarily explores women’s perceptions regarding the justification of domestic violence under different circumstances (Ewerling et al. 2017; Ewerling et al. 2020). The “social independence” domain comprises factors such as education, frequency of engagement with media (e.g., reading newspapers or magazines), age at first childbirth, age at the onset of cohabitation, and the differences in age and education between the woman and her partner. The third domain, “decision-making,” assesses the degree of a woman’s participation in key household decisions, providing insights into her autonomy within the home. Detailed methodology for developing the SWPER index has been described elsewhere and summarized here (Ewerling et al. 2017; Ewerling et al. 2020). The global SWPER index was derived using principal component analysis (PCA) based on 14 items extracted from 62 DHS surveys (list of the items is given in online supplemental Table 1) (Ewerling et al. 2020). The index was limited to women in partnerships, given the nature of some specific questions. For women without childbirth history, the age of first birth was inferred using the single hot-deck imputation method (Ewerling et al. 2020). Each item was weighted according to the PCA factor loadings to best represent each domain. The global SWPER scores were then categorized into tertiles as per the standard cut-off points suggested by the authors, dividing each SWPER domain into low, medium, or high levels of empowerment (Ewerling et al. 2020).

**Table 1:** Sociodemographic characteristics of the included participants from the 2022 Nepal Demographic and Health Survey

	Number (%)
No. of participants	5556
Mean age of women in years (SD)	32.50 (8.69)
Age groups	
15–29 years	2258 (40.6)
30 − 39 years	1904 (34.3)
40 − 49 years	1394 (25.1)
Area of residence	
Urban	2927 (52.7)
Rural	2629 (47.3)
Province	
Koshi	818 (14.7)
Madhesh	974 (17.5)
Bagmati	729 (13.1)
Gandaki	645 (11.6)
Lumbini	842 (15.2)
Karnali	766 (13.8)
Sudurpashchim	782 (14.1)
Highest educational level	
No education	1821 (32.8)
Primary	1888 (34.0)
Secondary	1692 (30.5)
Higher	155 (2.8)
Household’s wealth index	
Poorest	1539 (27.7)
Poorer	1119 (20.1)
Middle	1115 (20.1)
Richer	1048 (18.9)
Richest	735 (13.2)
Currently working	
No	1747 (31.4)
Yes	3809 (68.6)
Standardized attitude to violence SWPER score	0.67 (0.62, 0.72)
Standardized autonomy SWPER score	− 0.24 (− 0.73, 0.38)
Standardized decision-making SWPER score	− 0.47 (− 1.13, − 0.03)

*SWPER* Survey-Based Women’s Empowerment Index

### Outcome variables

In this study, we evaluated four outcomes: symptoms of anxiety, symptoms of depression, presence of any mental health symptoms, and seeking care for mental health issues. Symptoms of anxiety were measured using the Generalized Anxiety Disorder-7 scale (GAD-7) (Spitzer et al. 2006). Comprising seven items, the GAD-7 captures the primary characteristic of anxiety: persistent and debilitating worry. It also incorporates elements from three other prevalent anxiety disorders: panic disorder, social anxiety disorder, and post-traumatic stress disorder. Each item in the GAD-7 was scored from 0 to 3 based on the frequency of the symptom’s occurrence in the 2 weeks preceding the survey: 0 for “Never,” 1 for “Rarely,” 2 for “Often,” and 3 for “Always.” The sum of all item scores constituted the total GAD-7 score, which could range from 0 to 21. Respondents were classified as demonstrating anxiety symptoms if their GAD-7 score was 6 or more (Spitzer et al. 2006). Symptoms of depression were evaluated using the Patient Health Questionnaire (PHQ-9) (Kroenke et al. 2001). The PHQ-9, comprised of nine items, is based on the Diagnostic and Statistical Manual of Mental Disorders (DSM) criteria for depression diagnosis and is recognized as a reliable and valid measure of depression severity. Similar to the GAD-7, each PHQ-9 item was scored from 0 to 3 according to the frequency of the symptom’s occurrence in the preceding 2 weeks, and the scores were added to give the total PHQ-9 score. A PHQ-9 score could range from 0 to 27, and a score of 10 or above was considered indicative of depression symptoms (Kroenke et al. 2001). For assessing any mental health symptoms, respondents experiencing either anxiety or depression symptoms were identified as well as those taking medicine prescribed by a health care provider. Respondents who reported any symptoms of anxiety or depression in the 2 weeks preceding the survey (i.e., those with a score of 1 or more on either the GAD-7 or PHQ-9) were asked about whether they seek care for their symptoms (yes/no).

### Covariates

We included various sociodemographic characteristics in this study, including respondent’s age, place of residence, administrative province (Koshi, Madhesh, Bagmati, Gandaki, Lumbini, Karnali, Sudurpashchim), highest educational level of women, and household wealth index. Age was categorized into 15–29 years, 30–39 years, and 40–49 years. The definitions of rural and urban residences were guided by country-specific parameters. The socioeconomic status (SES) of the household was derived from the 2022 NDHS household wealth index, which was calculated using principal components analysis based on quantity and variety of consumer goods they own and their housing characteristics, such as source of drinking water, toilet facilities, and flooring materials. Wealth index was then assigned to each household member and used to divide the population into national wealth quintiles, each containing 20% of the population, from poorest (Q1) to richest (Q5) (Ministry of Health and Population [Nepal] New ERA and ICF 2023).

### Statistical analysis

We performed statistical analysis in accordance with the DHS guide to analyze DHS data (USAID), utilizing Stata v16.1 software and taking into account the complex survey design with Stata’s “svy” command. We used descriptive statistics to estimate proportions for categorical variables and means and standard deviations for continuous variables in our sample. We estimated the prevalence of symptoms of anxiety, symptoms of depression, any mental health symptoms, and care-seeking behavior according to various sociodemographic factors and SWPER domains. Chi-square tests were used to investigate the bivariate relationships between sociodemographic factors and mental health outcomes. We utilized both simple and multiple logistic regressions to investigate the associations of the three SWPER domains with the mental health outcomes. For multiple logistic regression, we included all covariates with *p*-values < 0.10 in bivariate analysis with the outcomes. Although educational level had *p*-value < 0.10, we did not include it in the final model because SWPER domains included educational level. The final regression models were adjusted for age, area of residence, provinces, and household wealth index and adjusted odds ratios (aORs) with 95% confidence intervals (CIs) were estimated. We considered an alpha level (α) of 0.05 as the cut-off for statistical significance, and all statistical tests were two-sided.

## Results

### Participant characteristics

Our analysis included a total of 5556 women from the 2022 NDHS. Table 1 illustrates the sociodemographic characteristics of the women included in the study. The average age of the participants was 32.5 years (SD 8.69) with a slight majority (52.7%) residing in urban areas. Geographical distribution shows participants spread across all provinces, from Gandaki (11.6%) to Madhesh (17.5%). The levels of education varied substantially, with 32.8% of the women having no education and only 2.8% with higher education. More than two-thirds (68.6%) of women were employed during the survey.

Utilizing the SWPER index, we found the standardized score for the “attitude to violence” domain to be 0.67 (0.62, 0.71), for the “social independence” domain − 0.24 (− 0.73, 0.38), and for the “decision-making” domain − 0.47 (− 1.13, − 0.03) (Table 1). A majority of women (80.7%) were categorized as highly empowered in the “attitude to violence” domain. Conversely, fewer women were categorized as highly empowered in the “social independence” (27.1%) and “decision-making” (9.1%) domains. Table 2 provides a detailed view of women’s empowerment levels according to various sociodemographic characteristics. Older women were less likely to demonstrate high empowerment in the “attitude to violence” and “social independence” domains, yet they were more likely to be highly empowered in the “decision-making” domain. Women living in rural areas generally exhibited lower levels of empowerment in the “social independence” and “decision-making” domains compared to their urban counterparts. Furthermore, women with no education or primary education exhibited lower levels of empowerment across all domains. However, higher education was strongly associated with high empowerment levels in the “social independence” domain. Women from wealthier households, particularly those in the richest quintile, demonstrated higher levels of empowerment across all domains (Table 2).

**Table 2:** Level of women empowerment by various sociodemographic characteristics

	Level of empowerment								
	Attitude to violence			Social independence			Decision-making		
	Low	Medium	High	Low	Medium	High	Low	Medium	High
**Overall**	**155 (2.8)**	**917 (16.5)**	**4484 (80.7)**	**1868 (15.6)**	**2182 (39.3)**	**1506 (27.1)**	**1503 (27.1)**	**3546 (63.8)**	**507 (9.1)**
Age groups									
15–29 years	52 (33.5)	399 (43.5)	1807 (40.3)	546 (29.2)	1001 (45.9)	711 (47.2)	881 (58.6)	1269 (35.8)	108 (21.3)
30–39 years	49 (31.6)	317 (34.6)	1538 (34.3)	702 (37.6)	662 (30.3)	540 (35.9)	334 (22.2)	1336 (37.7)	234 (46.2)
40–49 years	54 (34.8)	201 (21.9)	1139 (25.4)	620 (33.2)	519 (23.8)	255 (16.9)	288 (19.2)	941 (26.5)	165 (32.5)
Area of residence									
Urban	76 (49.0)	499 (54.4)	2352 (52.5)	920 (49.3)	1082 (49.6)	925 (61.4)	755 (50.2)	1894 (53.4)	278 (54.8)
Rural	79 (51.0)	418 (45.6)	2132 (47.5)	948 (50.7)	1100 (50.4)	581 (38.6)	748 (49.8)	1652 (46.6)	229 (45.2)
Province									
Koshi	22 (14.2)	160 (17.4)	636 (14.2)	194 (10.4)	328 (15.0)	296 (19.7)	171 (11.4)	560 (15.8)	87 (17.2)
Madhesh	33 (21.3)	104 (11.3)	837 (18.7)	534 (28.6)	332 (15.2)	108 (7.2)	414 (27.5)	484 (13.6)	76 (15.0)
Bagmati	21 (13.5)	110 (12.0)	598 (13.3)	193 (10.3)	257 (11.8)	279 (18.5)	144 (9.6)	507 (14.3)	78 (15.4)
Gandaki	9 (5.8)	94 (10.3)	542 (12.1)	156 (8.4)	263 (12.1)	226 (15.0)	95 (6.3)	468 (13.2)	82 (16.2)
Lumbini	21 (13.5)	155 (16.9)	666 (14.9)	253 (13.5)	351 (16.1)	238 (15.8)	251 (16.7)	513 (14.5)	78 (15.4)
Karnali	38 (24.5)	158 (17.2)	570 (12.7)	265 (14.2)	337 (15.4)	164 (10.9)	200 (13.3)	515 (14.5)	51 (10.1)
Sudurpashchim	11 (7.1)	136 (14.8)	635 (14.2)	273 (14.6)	314 (14.4)	195 (12.9)	228 (15.2)	499 (14.1)	55 (10.8)
Highest educational level									
No education	67 (43.2)	277 (30.2)	1477 (32.9)	1198 (64.1)	518 (23.7)	105 (7.0)	523 (34.8)	1110 (31.3)	188 (37.1)
Primary	54 (34.8)	349 (38.1)	1485 (33.1)	632 (33.8)	989 (45.3)	267 (17.7)	517 (34.4)	1186 (33.4)	185 (36.5)
Secondary	32 (20.6)	280 (30.5)	1380 (30.8)	38 (2.0)	667 (30.6)	987 (65.5)	440 (29.3)	1131 (31.9)	121 (23.9)
Higher	2 (1.3)	11 (1.2)	142 (3.2)	0 (0.0)	8 (0.4)	147 (9.8)	23 (1.5)	119 (3.4)	13 (2.6)
Household's wealth index									
Poorest	60 (38.7)	271 (29.6)	1208 (26.9)	576 (30.8)	708 (32.4)	255 (16.9)	402 (26.7)	1025 (28.9)	112 (22.1)
Poorer	39 (25.2)	201 (21.9)	879 (19.6)	446 (23.9)	427 (19.6)	246 (16.3)	329 (21.9)	689 (19.4)	101 (19.9)
Middle	30 (19.4)	175 (19.1)	910 (20.3)	435 (23.3)	415 (19.0)	265 (17.6)	331 (22.0)	660 (18.6)	124 (24.5)
Richer	14 (9.0)	176 (19.2)	858 (19.1)	301 (16.1)	420 (19.2)	327 (21.7)	294 (19.6)	651 (18.4)	103 (20.3)
Richest	12 (7.7)	94 (10.3)	629 (14.0)	110 (5.9)	212 (9.7)	413 (27.4)	147 (9.8)	521 (14.7)	67 (13.2)
Currently working									
No	35 (22.6)	313 (34.1)	1399 (31.2)	519 (27.8)	718 (32.9)	510 (33.9)	600 (39.9)	1027 (29.0)	120 (23.7)
Yes	120 (77.4)	604 (65.9)	3085 (68.8)	1349 (72.2)	1464 (67.1)	996 (66.1)	903 (60.1)	2519 (71.0)	387 (76.3)

^*^Country sampling weights provided by the Demographic and Health Survey and Stata’s survey estimation procedures were used to estimate country- representative estimates

### Prevalence of mental health symptoms and care-seeking behavior

Table 3 presents the percentage distribution of the seven symptoms associated with the GAD-7 scale (anxiety) and the nine symptoms associated with the PHQ-9 scale (depression). According to the GAD-7 scale, 23.1% of the women in our study exhibited symptoms of anxiety, with the highest prevalence of 26.0% observed in the 40–49 years age group (Table 4). The highest prevalence of anxiety (30.5%) was reported among women living in the Karnali province, while those in Bagmati province had the lowest prevalence (17.1%). Prevalence of anxiety was found to decrease with higher education levels (25.7% for those with no education vs. 14.8% for those with higher education). The lowest prevalence of anxiety was observed among those from the wealthiest households (16.3% compared to 25.0% in the poorest households). Conversely, 6.1% of the women showed symptoms of depression, with no significant differences across age groups. The highest prevalence of depression symptoms was found in Karnali (9.7%) and the lowest in Bagmati (3.7%). It was observed that women with higher education and those from wealthier households were less likely to experience depression symptoms (Table 4). The overall prevalence of any mental health symptoms was 23.5%. Among the women who experienced any mental health symptoms, 18.3% sought care. Care-seeking behavior was less prevalent among older women, those residing in rural areas, those with no education, and those from the poorest households (Table 4).

**Table 3:** Distributions of symptoms of anxiety and depression in the 2 weeks preceding the 2022 Nepal Demographic and Health Survey

	*N* (%)			
	Never	Rarely	Often	Always
Symptoms of anxiety				
Feeling nervous, anxious, or on edge	2757 (49.6)	2065 (37.2)	526 (9.5)	208 (3.7)
Not being able to stop or control worrying	4207 (75.7)	985 (17.7)	274 (4.9)	90 (1.6)
Worrying too much about different things	2970 (53.5)	1927 (34.7)	508 (9.1)	151 (2.7)
Trouble relaxing	3803 (68.4)	1316 (23.7)	330 (5.9)	107 (1.9)
Being so restless that it is hard to sit still	4291 (77.2)	939 (16.9)	257 (4.6)	69 (1.2)
Becoming easily annoyed or irritable	2517 (45.3)	2314 (41.6)	572 (10.3)	152 (2.7)
Feeling afraid as if something awful might happen	3640 (65.5)	1487 (26.8)	344 (6.2)	84 (1.5)
Symptom of depression				
Little interest or pleasure in doing things	3967 (71.4)	1172 (21.1)	326 (5.9)	91 (1.6)
Feeling down, depressed, or hopeless	3656 (65.8)	1442 (26.0)	368 (6.6)	90 (1.6)
Trouble falling asleep or staying asleep or sleeping too much	3790 (68.2)	1336 (24.0)	324 (5.8)	106 (1.9)
Feeling tired or having little energy	3404 (61.3)	1639 (29.5)	403 (7.3)	110 (2.0)
Poor appetite or overeating	3942 (71.0)	1290 (23.2)	261 (4.7)	63 (1.1)
Feeling bad about yourself or that you are a failure or have let yourself or your family down	4841 (87.1)	547 (9.8)	133 (2.4)	35 (0.6)
Trouble concentrating on things such as reading the newspaper or watching television	4453 (80.1)	870 (15.7)	194 (3.5)	39 (0.7)
Moving or speaking so slowly that other people could have noticed or the opposite	4946 (89.0)	476 (8.6)	112 (2.0)	22 (0.4)
Thoughts that you would be better off dead or of hurting yourself in some way	5124 (92.2)	333 (6.0)	77 (1.4)	22 (0.4)

^*^Country sampling weights provided by the Demographic and Health Survey and Stata’s survey estimation procedures were used to estimate country- representative estimates

**Table 4:** Prevalence of symptoms of anxiety, depression, any mental health problems, and proportion of care seeking, by various sociodemographic factors and SWPER domains

	Symptoms of anxiety		Symptoms of depression		Any mental health symptoms		Care seeking	
	*n* (%)	*P*-value	*n* (%)	*P*-value	*n* (%)	*P*-value	*n* (%)	*P*-value
Overall	1,283 (23.1)		340 (6.1)		1,307 (23.5)		825 (18.3)	
Age groups		0.011		0.33		0.006		0.19
15–29 years	508 (22.5)		141 (6.2)		520 (23.0)		351 (19.4)	
30–39 years	413 (21.7)		105 (5.5)		417 (21.9)		277 (18.2)	
40–49 years	362 (26.0)		94 (6.7)		370 (26.5)		197 (16.8)	
Area of residence		0.66		0.42		0.43		0.005
Urban	669 (22.9)		172 (5.9)		676 (23.1)		469 (19.9)	
Rural	614 (23.4)		168 (6.4)		631 (24.0)		356 (16.6)	
Province		< 0.001		< 0.001		< 0.001		0.005
Koshi	211 (25.8)		58 (7.1)		214 (26.2)		131 (18.7)	
Madhesh	218 (22.4)		58 (6.0)		224 (23.0)		149 (18.3)	
Bagmati	125 (17.1)		27 (3.7)		128 (17.6)		70 (12.5)	
Gandaki	120 (18.6)		25 (3.9)		121 (18.8)		85 (17.6)	
Lumbini	186 (22.1)		43 (5.1)		190 (22.6)		141 (21.1)	
Karnali	234 (30.5)		74 (9.7)		239 (31.2)		119 (18.4)	
Sudurpashchim	189 (24.2)		55 (7.0)		191 (24.4)		130 (20.5)	
Highest educational level		< 0.001		0.051		< 0.001		0.016
No education	468 (25.7)		117 (6.4)		474 (26.0)		250 (16.4)	
Primary	465 (24.6)		130 (6.9)		474 (25.1)		274 (17.9)	
Secondary	327 (19.3)		89 (5.3)		336 (19.9)		279 (21.0)	
Higher	23 (14.8)		4 (2.6)		23 (14.8)		22 (18.2)	
Household’s wealth index		< 0.001		0.004		< 0.001		< 0.001
Poorest	384 (25.0)		107 (7.0)		393 (25.5)		181 (14.2)	
Poorer	279 (24.9)		77 (6.9)		285 (25.5)		179 (19.7)	
Middle	280 (25.1)		74 (6.6)		283 (25.4)		182 (19.9)	
Richer	220 (21.0)		59 (5.6)		224 (21.4)		177 (20.7)	
Richest	120 (16.3)		23 (3.1)		122 (16.6)		106 (19.3)	
Currently working		0.18		0.54		0.20		0.41
No	384 (22.0)		112 (6.4)		392 (22.4)		245 (17.6)	
Yes	899 (23.6)		228 (6.0)		915 (24.0)		580 (18.6)	
Attitude to violence		0.50		0.61		0.62		0.29
Low	41 (26.5)		12 (7.7)		41 (26.5)		17 (13.1)	
Medium	218 (23.8)		59 (6.4)		220 (24.0)		141 (18.2)	
High	1,024 (22.8)		269 (6.0)		1,046 (23.3)		667 (18.5)	
Social independence		< 0.001		0.002		< 0.001		0.29
Low	495 (26.5)		143 (7.7)		503 (26.9)		280 (18.1)	
Medium	509 (23.3)		123 (5.6)		520 (23.8)		311 (17.5)	
High	279 (18.5)		74 (4.9)		284 (18.9)		234 (19.8)	
Decision-making		< 0.001		< 0.001		< 0.001		0.35
Low	345 (23.0)		103 (6.9)		353 (23.5)		218 (17.9)	
Medium	771 (21.7)		182 (5.1)		785 (22.1)		518 (18.1)	
High	167 (32.9)		55 (10.8)		169 (33.3)		89 (20.9)	

^*^Country sampling weights provided by the Demographic and Health Survey and Stata’s survey estimation procedures were used to estimate country-representative estimates

### Association between women empowerment and mental health outcomes

**Fig. 1:**
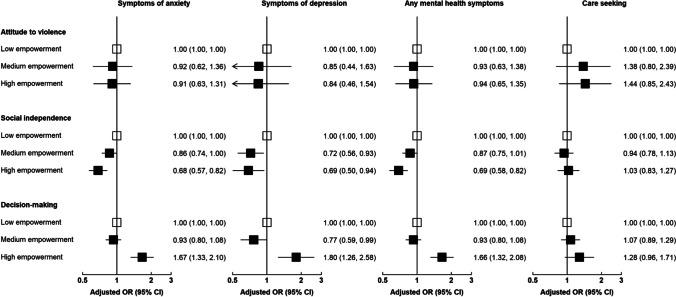
Associations of women empowerment domains with the prevalence of mental health symptoms and care-seeking behavior. Multiple logistic regressions were adjusted for age, province, area of residence, education level, employment status, and household wealth index. Odds ratios (ORs) are represented by squares, and their corresponding 95% CIs are represented by lines

Supplementary Table S2 shows the unadjusted ORs for the associations of women empowerment domains and mental health outcomes. The results from the multiple logistic regression with adjustment for sociodemographic factors showed a significant association between high empowerment in the “social independence” domain and lower odds of anxiety (aOR vs. low empowerment 0.68; 95% CI, 0.57–0.82), depression (aOR = 0.69; 95% CI, 0.50–0.94), and any mental health symptoms (aOR = 0.69; 95% CI, 0.58–0.82) (Fig. 1, Supplementary Table S3). Conversely, high empowerment in the “decision-making” domain was significantly associated with symptoms of depression (aOR = 1.67; 95% CI, 1.33–2.10), symptoms of depression (aOR = 1.80; 95% CI, 1.26–2.58), and any mental health symptoms (aOR = 1.66; 95% CI, 1.32–2.08). High level of empowerment in “decision-making” domain was also associated with higher likelihood of seeking care for mental health symptoms, but the association did not reach statistical significance (aOR = 1.28; 95% CI, 0.96–1.71). We observed no significant association between empowerment in the “attitude to violence” domain and mental health symptoms or care seeking (Fig. 1, Supplementary Table S3).

## Discussion

Our study investigated novel associations of various domains of women empowerment with mental health symptoms and care-seeking behavior in Nepal. We found that the overall prevalence of mental health problem was 23.5%, symptoms of anxiety 23.1%, and symptoms of depression 6.1%. Less than 1 in 5 women with symptoms of anxiety or depression sought care. Our study further revealed that compared to women with low level of empowerment in the “social independence” domain, those with high level of empowerment were less likely to suffer from symptoms of anxiety and depression. However, high empowerment in “decision-making” domain was associated with higher likelihood of anxiety and depression.

Our study highlights several important findings into the prevalence and care-seeking behavior for mental health problem among Nepalese women. We found that 23.1% and 6.1% of women exhibited symptoms of anxiety and depression, respectively. Comparatively, the 2022 NDHS data showed that these rates in men were 11% and 2%, indicating that women were approximately twice as likely to experience mental health problems as men (Ministry of Health and Population [Nepal] New ERA and ICF 2023). The recent National Mental Health Survey in Nepal, conducted in 2000, reported that the prevalence of any mental disorder—such as schizophrenia, bipolar disorder, major depressive disorder, or suicidal thoughts—among individuals aged 18 years and above was 10.0% for lifetime (Dhimal et al. 2022). On the other hand, 4.3% were currently suffering from these disorders, with women affected slightly more than men (5.1% vs. 3.4%) (Dhimal et al. 2022). According to Global Burden of Disease (GBD) estimates from 2019, women in Nepal had a higher prevalence of major depressive disorder compared to men (4.3% vs 2.8%) (Nepal 2022). While direct comparison of prevalence rates across these estimates from different studies can be challenging due to varying definitions of mental health problems, a clear pattern emerges: women in Nepal bear a disproportionately higher burden of mental health issues than men. A range of social and gender-specific factors determine the prevalence and trajectory of mental disorders among women. In many low- and middle-income countries (LMICs), social factors such as poverty, urbanization, internal migration, and lifestyle changes contribute to the high prevalence of mental illness (Rathod et al. 2017). Alongside, issues such as depression, anxiety, psychological distress, sexual violence, and domestic violence impact women more profoundly than men across various countries and cultures (Srivastava 2012). The cumulative pressures of women’s multiple roles, gender discrimination, and associated factors like domestic violence and sexual abuse coalesce to explain the poor mental health status of women (Srivastava 2012, Rathod et al. 2017) .

Our study revealed that only 18.3% of women experiencing symptoms of anxiety or depression sought care, mirroring low care-seeking rates in other LMICs (Rathod et al. 2017). However, it is important to mention that the NDHS includes data on care sought both from healthcare providers and alternative sources, which could inflate the reported rate of care-seeking for mental health issues in our study. Cultural and religious beliefs about mental health illness, which shape help-seeking behavior, often hamper access to mental health services. The prevailing stigma of mental illness exacerbates these obstacles and even leads to the growing popularity of non-evidence-based treatments. Other factors for lower care-seeking behavior among women include limited resources, both human and financial, and institutional biases against women and marginalized communities (Srivastava 2012, Malhotra and Shah 2015, Rathod et al. 2017). One recent study involving female community health volunteers in Nepal noted that the fear of discrimination and the ensuing shame often deterred individuals from seeking mental health treatment at health facilities, leading to high dropout rates (Upadhaya et al. 2020).

Our study highlighted that women with high empowerment in the “social independence” domain, as determined by the SWERP index, were less likely to exhibit mental health symptoms. The social independence domain is primarily constructed around prerequisites that empower women to achieve their goals. These factors include educational attainment, access to information, age at crucial life events like marriage or cohabitation, spousal asset differentials, and access to information (Ewerling et al. 2020). Given the parameters used to formulate this domain, it is plausible that women who are well-informed and did not have to halt their education or careers due to early life events like marriage or childbirth are less prone to mental health issues (Compton 2015). The correlation between women empowerment via income generation and education and enhanced individual and community mental health has been previously reported (Kermode et al. 2007; Leight et al. 2022).

Conversely, we observed that high empowerment in the “decision-making” domain corresponded with an increased likelihood of mental health symptoms. While previous literature indicated that women’s participation in decision-making can reduce mental health problems (Shooshtari et al. 2018), our findings of positive associations between empowerment in “decision-making” domain and mental health symptoms is surprising. The decision-making domain is defined by the respondent’s involvement in personal healthcare decisions, significant household purchases, and visits to family or relatives (Ewerling et al. 2020). This could suggest that women participating in familial decision-making processes might be pushing back against existing gender norms and societal expectations, leading to additional stress, and exacerbating their mental health conditions. A previous qualitative study from India highlighted conflict with husbands and mothers-in-law as the commonest mental health stressors for women (Kermode et al. 2007). Alternatively, highly empowered women might be more willing to disclose their mental health struggles, which could result in a reverse causation between high empowerment and mental health symptoms. It is worth mentioning that we found a trend towards increased care-seeking behavior among highly empowered women in the decision-making domain, although this association did not reach statistical significance.

The interplay between women empowerment and mental health outcomes in LMICs necessitates examination through cultural, gender, and psychological lenses. Culturally, many societies have historically confined women to subservient roles, suppressing their autonomy and decision-making. Such restrictions, amplified by cultural norms, can breed feelings of entrapment, hopelessness, and marginalization, thereby affecting mental health (Bhugra et al. 2021). From a gender perspective, the structural imbalances and systemic inequities women face magnify these mental health challenges. Factors like limited access to educational opportunities, economic resources, or even basic rights can lead to chronic stress, low self-esteem, and feelings of worthlessness, which can compound over time, leading to overt mental health problems like anxiety and depression (Seedat et al. 2009; Yu 2018). Psychologically, empowerment is not just about external freedoms but also encompasses internal emancipation (Ibsen 2023). It embodies a woman’s self-view, confidence in her abilities, and her influence over her environment. Empowered women are inherently more resilient, adept at navigating adversities, and proactively caring for their mental well-being (Sisto et al. 2019). Overall, empowerment acts as a buffer, offering women a protective layer against the myriad mental health challenges they might encounter in their lifetimes. Although previous studies showed relationships between empowerment and mental health (Shooshtari et al. 2018), our study seeks to extend the knowledge base by focusing on Nepalese women. This is particularly important given the unique cultural context of Nepal, which differs significantly from the settings of previous research. Our findings prompt society at large to acknowledge and facilitate women empowerment as a strategy for improving mental health among women living in Nepal as well as in LMICs.

The strengths of our study include utilization of standardized methodologies and the use of the globally recognized and validated SWPER index to measure women’s empowerment (Ewerling et al. 2017; Ewerling et al. 2020). Furthermore, we used nationally representative survey data with validated questionnaires for assessing mental health symptoms. Despite these strengths, our study also has several limitations. While robust, the SWPER index does not encompass all aspects of women’s empowerment, a complex construct encompassing economic, sociocultural, familial, interpersonal, legal, political, societal, and psychosocial facets (Anik et al. 2021, Wendt et al. 2022). The SWPER index also lacks information on personal asset ownership, economic participation, and involvement in governance processes (Ewerling et al. 2017). Additionally, our study population was limited to married or cohabitating women, so the findings may not be representative of all women. Despite adjusting for various sociodemographic factors, the possibility of residual confounding cannot be ruled out in the associations between women empowerment domains and the prevalence of mental health symptoms. Finally, it is essential to note that in LMICs such as Nepal, both men and women continue to face inadequate access to and utilization of mental health services. While our study suggests that women empowerment may not directly influence mental health care-seeking behaviors, various factors, including health literacy, sociocultural support, and healthcare resources, can also affect access to mental health care. Unfortunately, we could not study these aspects in our study.

In conclusion, our study highlighted a significant burden for symptoms of anxiety and depression and low level of care-seeking behavior for such symptoms among women in Nepal. We also showed that women considered as highly empowered in social independence domain were less likely to suffer from mental health symptoms. Our findings suggest a critical pathway through which women’s empowerment can improve their mental well-being. Therefore, it is important for health policies to accommodate women’s needs and foster their empowerment to alleviate the burden of mental health issues and promote greater access to relevant health services. Moreover, to enhance social independence and decision-making in women in LMICs, it is crucial to invest in their education, provide access to economic opportunities, challenge traditional gender norms through community engagement, and strengthen legal frameworks that uphold women’s rights.

### Supplementary Information

Below is the link to the electronic supplementary material.

Supplementary file1 (DOCX 23 KB)

## Data Availability

The 2022 Nepal Demographic and Health Survey dataset used in this study are publicly available at this link: https://dhsprogram.com/data/.

## References

[CR1] Anik AI, Ghose B, Rahman MM (2021). “Relationship between maternal healthcare utilisation and empowerment among women in Bangladesh: evidence from a nationally representative cross-sectional study. BMJ Open.

[CR2] Bhugra D, Watson C, Wijesuriya R (2021). Culture and mental illnesses. Int Rev Psychiatry.

[CR3] Compton MT (2015). The social determinants of mental health.

[CR4] Dhimal M, Dahal S, Adhikari K, Koirala P, Bista B, Luitel N, Pant S, Marahatta K, Shakya S, Sharma P, Ghimire S, Gyanwali P, Ojha SP, Jha AK (2022). A nationwide prevalence of common mental disorders and suicidality in nepal: evidence from National Mental Health Survey, 2019–2020. J Nepal Health Res Counc.

[CR5] Ewerling F, Lynch JW, Victora CG, van Eerdewijk A, Tyszler M, Barros AJD (2017). The SWPER index for women’s empowerment in Africa: development and validation of an index based on survey data. Lancet Glob Health.

[CR6] Ewerling F, Raj A, Victora CG, Hellwig F, Coll CV, Barros AJ (2020). SWPER Global: A Survey-Based Women’s Empowerment index expanded from Africa to all low- and middle-income countries. J Glob Health.

[CR7] Ibsen MF (2023). Domination, social norms, and the idea of an emancipatory interest. Constellations.

[CR8] Kermode M, Herrman H, Arole R, White J, Premkumar R, Patel V (2007). Empowerment of women and mental health promotion: a qualitative study in rural Maharashtra, India. BMC Public Health.

[CR9] Kroenke K, Spitzer RL, Williams JB (2001). The PHQ-9: validity of a brief depression severity measure. J Gen Intern Med.

[CR10] Leight J, Pedehombga A, Ganaba R, Gelli A (2022). Women’s empowerment, maternal depression, and stress: evidence from rural Burkina Faso. SSM Ment Health.

[CR11] Malhotra S, Shah R (2015). Women and mental health in India: an overview. Indian J Psychiatry.

[CR12] Ministry of Health and Population [Nepal] New ERA and ICF (2023). Nepal Demographic and Health Survey 2022. Kathmandu, Nepal, Ministry of Health and Population [Nepal].

[CR13] Nepal WHO (2022). Nepal WHO Special Initiative for Mental Health Situational Assessment

[CR14] Patel V (2007). Mental health in low- and middle-income countries. Br Med Bull.

[CR15] Rai Y, Gurung D, Gautam K (2021). Insight and challenges: mental health services in Nepal. Bjpsych Int.

[CR16] Rathod S, Pinninti N, Irfan M, Gorczynski P, Rathod P, Gega L, Naeem F (2017). “Mental health service provision in low- and middle-income countries. Health Serv Insights.

[CR17] Seedat S, Scott KM, Angermeyer MC, Berglund P, Bromet EJ, Brugha TS, Demyttenaere K, de Girolamo G, Haro JM, Jin R, Karam EG, Kovess-Masfety V, Levinson D, Medina Mora ME, Ono Y, Ormel J, Pennell BE, Posada-Villa J, Sampson NA, Williams D, Kessler RC (2009). Cross-national associations between gender and mental disorders in the World Health Organization World Mental Health Surveys. Arch Gen Psychiatry.

[CR18] Shooshtari S, Abedi MR, Bahrami M, Samouei R (2018). Empowerment of women and mental health improvement with a preventive approach. J Educ Health Promot.

[CR19] Sisto, A., F. Vicinanza, L. L. Campanozzi, G. Ricci, D. Tartaglini and V. Tambone (2019). “Towards a transversal definition of psychological resilience: a literature review.” Medicina (Kaunas) 55(11). 10.3390/medicina55110745 PMC6915594 31744109

[CR20] Spitzer RL, Kroenke K, Williams JB, Löwe B (2006). A brief measure for assessing generalized anxiety disorder: the GAD-7. Arch Intern Med.

[CR21] Srivastava K (2012). Women and mental health: psychosocial perspective. Ind Psychiatry J.

[CR22] United Nations Development Programme (2016). Human development report 2016.

[CR23] Upadhaya N, Regmi U, Gurung D, Luitel NP, Petersen I, Jordans MJD, Komproe IH (2020). Mental health and psychosocial support services in primary health care in Nepal: perceived facilitating factors, barriers and strategies for improvement. BMC Psychiatry.

[CR24] USAID. “The Demographic and Health Surveys (DHS) Program.” Retrieved 2 July 2023 from https://dhsprogram.com/.

[CR25] Walker, L., I. Verins, R. Moodie and K. J. P. M. H. Webster (2005). “Responding to the social and economic determinants of mental health: a conceptual framework for action.” 89–108.

[CR26] Wendt A, Santos TM, Cata-Preta BO, Costa JC, Mengistu T, Hogan DR, Victora CG, Barros AJD (2022). Children of more empowered women are less likely to be left without vaccination in low- and middle-income countries: a global analysis of 50 DHS surveys. J Glob Health.

[CR27] UN Women. “Gender data gaps and country performance: Nepal.” Retrieved 2 July 2023, from https://data.unwomen.org/country/nepal.

[CR28] Yu S (2018). Uncovering the hidden impacts of inequality on mental health: a global study. Transl Psychiatry.

