# Supplementary material for: Role of women empowerment on mental health problems and care-seeking behavior among married women in Nepal: secondary analysis of nationally representative data
Source: Arch Womens Ment Health. 2024 Feb 5;27(4):527–36. doi: 10.1007/s00737-024-01433-5 (PMC11230993; doi:10.1007/s00737-024-01433-5)
Supplement: Supplementary file 1 — Supplementary file1 (DOCX 23 KB) [file 737_2024_1433_MOESM1_ESM.docx]

**Role of women empowerment on mental health problems and care seeking behavior among married women in Nepal: secondary analysis of nationally representative data**

**Supplementary materials**

**Table S1: Items used in the development of the survey-based women’s empowerment index**

| **Survey items** | **Code or unit** |
| --- | --- |
| Beating not justified if wife goes out without telling husband | Justified=–1; don’t know=0; not justified =1 |
| Beating not justified if wife neglects the children | Justified=–1; don’t know=0; not justified =1 |
| Beating not justified if wife argues with husband | Justified=–1; don’t know=0; not justified =1 |
| Beating not justified if wife refuses to have sex with husband | Justified=–1; don’t know=0; not justified =1 |
| Beating not justified if wife burns the food | Justified=–1; don’t know=0; not justified =1 |
| Frequency of reading newspaper or magazine | Not at all=0; <once a week=1; ≥once a week=2 |
| Woman’s education in completed years of schooling | Years |
| Education difference: woman’s minus husband’s completed years of schooling | Years |
| Age difference: woman’s age minus husband’s age | Years |
| Age at first cohabitation | Years |
| Age of woman at first birth* | Years |
| Who usually decides on respondent’s health care | Husband or other alone=–1; joint=0; respondent alone=1 |
| Who usually decides on large household purchases | Husband or other alone=–1; joint=0; respondent alone=1 |
| Who usually decides on visits to family or relatives | Husband or other alone=–1; joint=0; respondent alone=1 |
| * Imputed for women who had not had a child.  Adapted from: Ewerling F, Lynch JW, Victora CG, van Eerdewijk A, Tyszler M, Barros AJD. The SWPER index for women's empowerment in Africa: development and validation of an index based on survey data. Lancet Glob Health. 2017 Sep;5(9):e916-e923. doi: 10.1016/S2214-109X(17)30292-9. Epub 2017 Jul 26. PMID: 28755895; PMCID: PMC5554795. | |

**Table S2: Unadjusted odds ratio between domains of women empowerment and mental health outcomes**

|  | **Symptoms of anxiety** | | **Symptoms of depression** | | **Any mental health symptoms** | | **Care seeking** | |
| --- | --- | --- | --- | --- | --- | --- | --- | --- |
|  | **Unadjusted OR (95% CI)** | **p-value** | **Unadjusted OR (95% CI)** | **p-value** | **Unadjusted OR (95% CI)** | **p-value** | **Unadjusted OR (95% CI)** | **p-value** |
| Attitude to violence |  |  |  |  |  |  |  |  |
| Low empowerment | 1.00 (Ref) |  | 1.00 (Ref) |  | 1.00 (Ref) |  | 1.00 (Ref) |  |
| Medium empowerment | 0.87 (0.59-1.28) | 0.471 | 0.82 (0.43-1.56) | 0.545 | 0.88 (0.60-1.29) | 0.509 | 1.48 (0.86-2.54) | 0.157 |
| High empowerment | 0.82 (0.57-1.18) | 0.293 | 0.76 (0.42-1.39) | 0.373 | 0.85 (0.59-1.22) | 0.367 | 1.51 (0.90-2.54) | 0.117 |
|  |  |  |  |  |  |  |  |  |
| Social independence |  |  |  |  |  |  |  |  |
| Low empowerment | 1.00 (Ref) |  | 1.00 (Ref) |  | 1.00 (Ref) |  | 1.00 (Ref) |  |
| Medium empowerment | 0.84 (0.73-0.97) | 0.020 | 0.72 (0.56-0.92) | 0.010 | 0.85 (0.74-0.98) | 0.024 | 0.96 (0.80-1.15) | 0.651 |
| High empowerment | 0.63 (0.53-0.74) | <0.001 | 0.62 (0.47-0.83) | 0.001 | 0.63 (0.53-0.74) | <0.001 | 1.11 (0.92-1.35) | 0.277 |
|  |  |  |  |  |  |  |  |  |
| Decision-making |  |  |  |  |  |  |  |  |
| Low empowerment | 1.00 (Ref) |  | 1.00 (Ref) |  | 1.00 (Ref) |  | 1.00 (Ref) |  |
| Medium empowerment | 0.93 (0.81-1.08) | 0.343 | 0.74 (0.57-0.94) | 0.016 | 0.93 (0.80-1.07) | 0.294 | 1.02 (0.85-1.21) | 0.858 |
| High empowerment | 1.65 (1.32-2.06) | <0.001 | 1.65 (1.17-2.33) | 0.004 | 1.63 (1.31-2.03) | <0.001 | 1.21 (0.92-1.60) | 0.170 |
|  |  |  |  |  |  |  |  |  |

**Table S3: Adjusted odds ratio between domains of women empowerment and mental health outcomes**

|  | **Symptoms of anxiety** | | **Symptoms of depression** | | **Any mental health symptoms** | | **Care seeking** | |
| --- | --- | --- | --- | --- | --- | --- | --- | --- |
|  | **Adjusted OR (95% CI)** | **p-value** | **Adjusted OR (95% CI)** | **p-value** | **Adjusted OR (95% CI)** | **p-value** | **Adjusted OR (95% CI)** | **p-value** |
| Attitude to violence |  |  |  |  |  |  |  |  |
| Low empowerment | 1.00 (Ref) |  | 1.00 (Ref) |  | 1.00 (Ref) |  | 1.00 (Ref) |  |
| Medium empowerment | 0.92 (0.62-1.36) | 0.665 | 0.85 (0.44-1.63) | 0.622 | 0.93 (0.63-1.38) | 0.724 | 1.38 (0.80-2.39) | 0.245 |
| High empowerment | 0.91 (0.63-1.31) | 0.607 | 0.84 (0.46-1.54) | 0.573 | 0.94 (0.65-1.35) | 0.727 | 1.44 (0.85-2.43) | 0.170 |
|  |  |  |  |  |  |  |  |  |
| Social independence |  |  |  |  |  |  |  |  |
| Low empowerment | 1.00 (Ref) |  | 1.00 (Ref) |  | 1.00 (Ref) |  | 1.00 (Ref) |  |
| Medium empowerment | 0.86 (0.74-1.00) | 0.049 | 0.72 (0.56-0.93) | 0.013 | 0.87 (0.75-1.01) | 0.060 | 0.94 (0.78-1.13) | 0.504 |
| High empowerment | 0.68 (0.57-0.82) | <0.001 | 0.69 (0.50-0.94) | 0.019 | 0.69 (0.58-0.82) | <0.001 | 1.03 (0.83-1.27) | 0.804 |
|  |  |  |  |  |  |  |  |  |
| Decision-making |  |  |  |  |  |  |  |  |
| Low empowerment | 1.00 (Ref) |  | 1.00 (Ref) |  | 1.00 (Ref) |  | 1.00 (Ref) |  |
| Medium empowerment | 0.93 (0.80-1.08) | 0.358 | 0.77 (0.59-0.99) | 0.044 | 0.93 (0.80-1.08) | 0.333 | 1.07 (0.89-1.29) | 0.458 |
| High empowerment | 1.67 (1.33-2.10) | <0.001 | 1.80 (1.26-2.58) | 0.001 | 1.66 (1.32-2.08) | <0.001 | 1.28 (0.96-1.71) | 0.090 |
|  |  |  |  |  |  |  |  |  |
